# The Approaching Meeting of the State Medical Association

**Published:** 1883-04

**Authors:** 


### THE APPROACHING MEETING OF THE STATE MEDICAL ASSOCIATION.

For the first time in the history of this organization, it will meet in the city of Athens, which is one of the most attractive cities of the State. The body will be called to order by the retiring President, Dr. William F. Holt, of Macon, April 18th, and the session will last three days. After the preliminary exercises, the chair will be assumed by the President elect, Dr. K. P. Moore, of Forsyth.

It is for obvious reasons, extremely desirable, that every reputable medical man within the State, should be a member of this Association, and, as far as possible, a regular attendant upon its sessions. Apart from the direct benefit conferred upon participants in the ordinary proceedings, the recreation incident to these meetings, and the social features of the reunions, are by no means unworthy of consideration. Life is short, and sometimes hard, but it need not always of necessity be made up entirely of weary, unrequited toil.

We know very well that it is oft-times inconvenient for medical men, engaged in an active and exacting practice, to leave their work and repair to the place selected for the meeting, especially if it be located in a remote part of the State; but even at a sacrifice of important business for a few days, we believe the indirect benefits derived from attendance upon these sessions will, in most instances, more than compensate the temporary inconvenience and loss.

Let us have at Athens a large and enthusiastic gathering. Let us emulate the worthy examples of our sister States, and cultivate our State society. If every man will only "act well his part," the result will surely be all that can reasonably be asked, and the benefits that will certainly accrue will prove a generous return for the outlay, both to the profession as a body, and to the individual members thereof.

